# A Review on the Effect of Fabric Reinforcement on Strength Enhancement of Natural Fiber Composites

**DOI:** 10.3390/ma15093025

**Published:** 2022-04-21

**Authors:** Soundhar Arumugam, Jayakrishna Kandasamy, Subramani Venkatesan, Rajesh Murugan, Valayapathy Lakshmi Narayanan, Mohamed Thariq Hameed Sultan, Farah Syazwani Shahar, Ain Umaira Md Shah, Tabrej Khan, Tamer Ali Sebaey

**Affiliations:** 1 Department of Mechanical Engineering, Indian Institute of Technology, Guwahati 781039, India; soundhar1372214@gmail.com; 2 School of Mechanical Engineering, Vellore Institute of Technology, Vellore 632014, India; venkatesans@vit.ac.in (S.V.); rajesh.murugan@vit.ac.in (R.M.); lakshmi.narayan2017@vitstudent.ac.in (V.L.N.); 3 Laboratory of Biocomposite Technology, Institute of Tropical Forestry and Forest Products (INTROP), Universiti Putra Malaysia, Serdang, Seri Kembangan 43400, Selangor Darul Ehsan, Malaysia; 4 Aerospace Malaysia Innovation Centre (944751-A), Prime Minister’s Department, MIGHT Partnership Hub, Jalan Impact, Cyberjaya 63000, Selangor Darul Ehsan, Malaysia; 5 Department of Aerospace Engineering, Faculty of Engineering, Universiti Putra Malaysia, UPM Serdang, Seri Kembangan 43400, Selangor Darul Ehsan, Malaysia; farahsyaz93@yahoo.com (F.S.S.); ainumaira91@gmail.com (A.U.M.S.); 6 Engineering Management Department, College of Engineering, Prince Sultan University, Riyadh, Saudi Arabia; tkhan@psu.edu.sa; 7 Mechanical Design and Production Department, Faculty of Engineering, Zagazig University, Zagazig, Sharkia, Egypt

**Keywords:** natural fiber composite, woven natural fiber, orientation, mechanical, dynamic mechanical, vibration

## Abstract

The main objective of this study is to examine the impact of reinforcements on the strength of natural fiber composites. Recent advancements in natural fiber composites have minimized the usage of man-made fibers, especially in the field of structural applications such as aircraft stiffeners and rotor blades. However, large variations in the strength and modulus of natural fiber degrade the properties of the composites and lower the safety level of the structures under dynamic load. Without compromising the safety of the composite structure, it is significant to enrich the strength and modulus of natural fiber reinforcement for real-time applications. The strength and durability of natural fiber can be enriched by reinforcing natural fiber. The reinforcement effect on natural fiber in their woven, braided, and knit forms enhances their structural properties. It improves the properties of natural fiber composites related to reinforcement with short and random-orientation fibers. The article also reviews the effect of the hybridization of natural fiber with cellulosic fiber, synthetic fiber, and intra-ply hybridization on its mechanical properties, dynamic mechanical properties, and free vibration characteristics, which are important for predicting the life and performance of natural fiber composites for weight-sensitive applications under dynamic load.

## 1. Introduction

**Figure 1:**
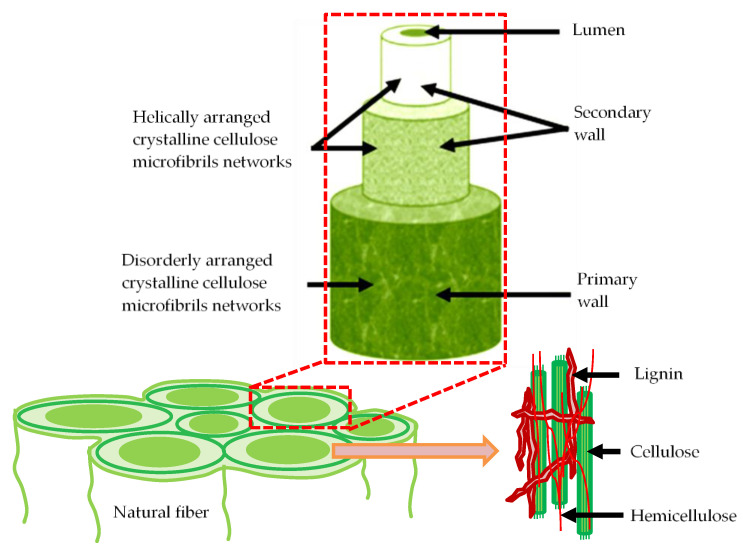
Structure of natural fiber.

This century has already perceived notable achievements in green technology, especially in the domain of materials science, with the evolution of high-performance materials made from natural resources for various structural, manufacturing, bio-medical, aerospace, and automotive applications [1,2]. Due to their natural abundance, ease of processing, design flexibility, and feasibility of manufacturing complex shapes, natural fiber composites are good alternatives to conventional materials. They are also light in weight and hazardless to the environment [3,4]. The main problem with natural fibers is the variation of properties and characteristics, such as strength and modulus [5]. The cellulose composition of the cell wall, environmental circumstances during growth, geographical considerations, microfibrillar angle, and other factors all influence the fiber’s strength [6]. The structure of the natural fiber is shown in Figure 1. Properties of natural fiber composites depend on several parameters, such as processing technique, fiber strength, interfacial bonding between fiber and matrix, type of reinforcement, weaving pattern, and fiber orientation [7].

**Figure 2:**
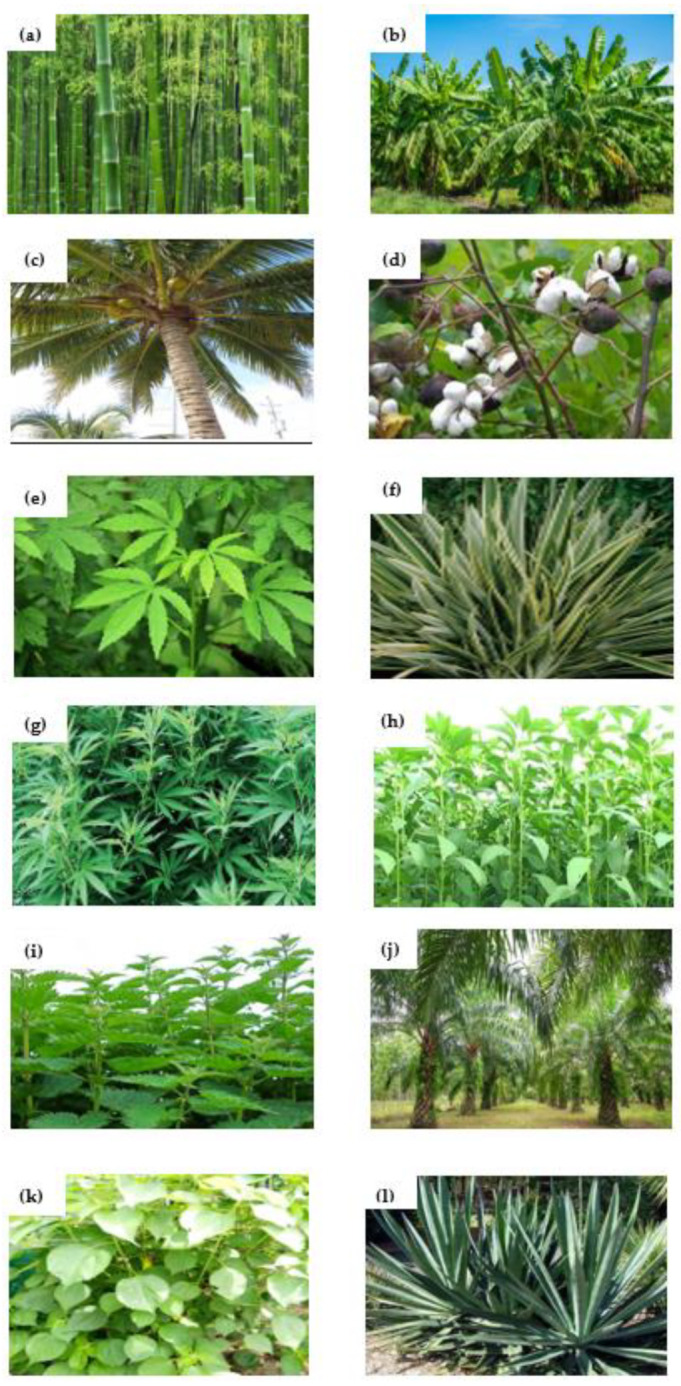
Common natural fibers used in composites: (**a**) bamboo (grass fiber type); (**b**) banana (leaf fiber type); (**c**) coir (fruit fiber type); (**d**) cotton (seed fiber type); (**e**) kenaf (bast fiber type); (**f**) flax (bast fiber type); (**g**) hemp (bast fiber type); (**h**) jute (bast fiber type); (**i**) nettle (grass fiber type); (**j**) oil palm (fruit fiber type); (**k**) ramie (bast fiber type); (**l**) sisal (leaf fiber type).

**Figure 3:**
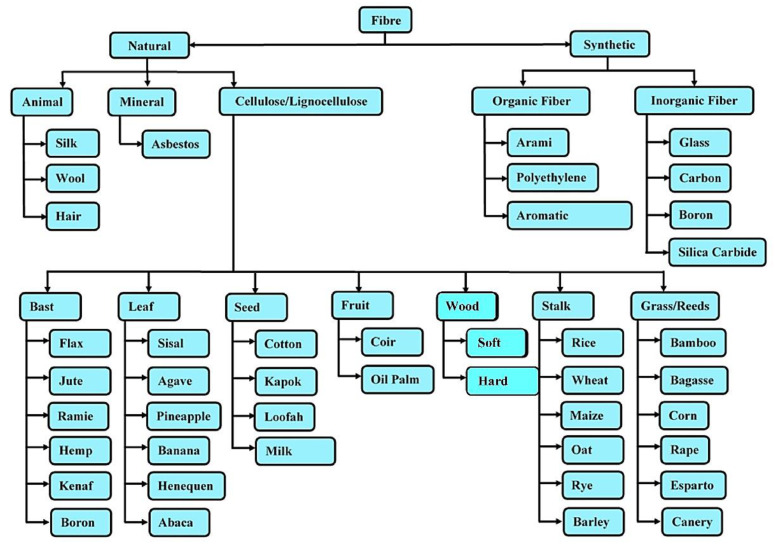
Classification of natural fibers.

In the last two decades, natural fiber has found extensive usage in aerospace, naval, and structural fields [8]. The main reasons behind the higher usage of natural fiber composites for weight-sensitive and structural applications such as stiffener, truss members, bio-medical, automobile interior parts, etc., by industries are the potential environmental, end-of-life discarding, and health advantages in contrast to man-made composite materials [9]. Furthermore, man-made composite materials have a high density, are expensive, and are hazardous to the environment compared to natural fiber composites [10]. As proof, natural fibers such as jute, flax, banana, hemp, and sisal have replaced synthetic fibers such as glass, carbon, Kevlar, and boron in fields that require a load-carrying capacity in the medium and low range [11]. Tarasen and Reddy [12] established the usage of natural fibers (bamboo and jute) in several areas, such as fiber-reinforced columns, special joints, packaging material, and pillars. Moreover, some natural fibers are the best sources for extracting nanocellulose fibers. These nanofibers can be added as a second reinforcement to naturally based composites. Figure 2 depicts different natural fibers utilized in composites, and Figure 3 displays the classification of natural fibers. Moreover, composites consist of two phases: One is the matrix phase and the other is the reinforcement phase. The reinforcement phase consists of lignocellulose, which is generally referred to as natural fiber composite. The fibers directly extracted from a living organism are called natural fibers. The fibers derived from synthetic materials are called synthetic fibers.

The large variation in strength and modulus makes NFCs incompatible under dynamic load and minimizes the safety level of the component. To overcome this drawback, NFCs are reinforced with NFRs in the polymer matrix to improve the structural applications’ strength, modulus, and safety.

Most researchers have developed NFCs with random orientation and short natural fibers as reinforcements in the polymer matrix, creating non-uniform stress distribution due to fiber discontinuity, which further leads to early failure of composites. The modulus of natural fibers can be enriched by reinforcing them with natural fibers in plain, braided, and knitted arrangements. It is observed that the Young’s modulus of NFCs reinforced with NFRs in distinctive patterns in weavings such as basket, plain, stain, twill, etc. have increased promisingly. Similarly, braided NRCs enriched the Young’s modulus of jute-fiber reinforcement by 30% compared to conventional weaving [12,13]. Sapuan and Maleque [13] developed less expensive telephone stands using banana fabric (woven type) in an epoxy matrix. By substituting fiberglass with jute fiber composites, Alves et al. [14] highlighted the advantages of NFCs in the manufacture of automobile hoods.

Reinforcing the NFC with synthetic fiber enhances its mechanical properties and load-carrying capacities and can be used for different structural applications [15]. Damodaran et al. [16] applied a basic sandwich model to develop a traditional drum (Chenda) using a carbon epoxy composite and balsa core material. Comparing the acoustic performance of the traditional drum and a composite drum suggested that the high damping properties of sandwich composites could replace the wood used in the traditional drum. Based on the mechanical properties, many researchers are focusing on working on identifying fibers (plant-based) suitable for use in medium and low load applications [17,18,19,20,21,22,23,24]. The benefits of woven fabric natural composites (WFNCs) have led to an increase in their use in a variety of structural applications. When compared to randomly oriented and unidirectional NFCs, WFNCs provide higher stiffness and strength for the same amount of fibers employed. NFCs’ fracture toughness is also improved by the usage of woven fabric. Riedel et al. studied the usage of WFNCs in several structural applications and concluded that using woven fabric would improve composite stiffness [25].

In this paper, a systematic review of NFC enhancement by modifying the Young’s modulus of natural fiber reinforcement is reported. A critical review was done on various processes such as weaving, braiding, and knitting concerning mechanical, dynamic mechanical, and free vibration properties. Further hybridization effects of natural fiber with synthetic fiber in the context of mechanical, dynamic mechanical, and dynamic properties were reviewed. In addition, the fabrication and advantages of intra-ply woven NFC made with two different yarn fibers are also discussed.

## 2. Disadvantages of Composites including Short Natural Fibers

Many researchers have investigated the mechanical, dynamic mechanical, and tribological properties of randomly oriented and short natural fibers as reinforcements in the polymer matrix. The main problem associated with short-form reinforcement in a high-density polymer matrix is that achieving uniform distribution is difficult. It affects the advantages of natural fiber composites seriously and makes them incompatible for structural applications. Almeida et al. [26] investigated the mechanical characteristics of coir fiber in a polyester matrix with a fraction of up to 80 wt% coir fiber. They found that composites with 50 wt% exhibited enhanced mechanical properties. Further addition resulted in less strength and a lower modulus of the composites due to random distribution and poor bonding between the fiber and matrix.

**Figure 4:**
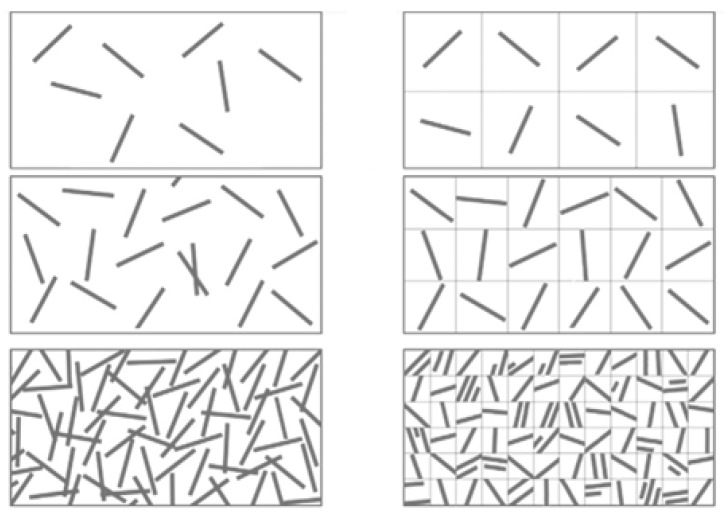
Schematic diagram of randomly oriented short fiber composite.

Another problem associated with randomly distributed short natural fibers is that the polymer matrix is agglomerates as it affects the composites properties. A similar problem was reported by Joseph et al. [27] regarding the mechanical properties of sisal/polypropylene composites. The authors concluded that fiber length, loading, and orientation affect the performance of the composites. A schematic diagram of a randomly oriented short fiber-reinforced composite is illustrated in Figure 4.

Arib et al. [28] studied the mechanical properties of pineapple leaf fiber-reinforced polypropylene composites and found that a higher volume percentage diminished the mechanical properties of the pineapple composites. Shekeil et al. [29] investigated the mechanical characteristics of kenaf fiber–thermoplastic polyurethane composites as a function of fiber weight %. They discovered that adding 30 wt% to the composites enhanced the mechanical characteristics of the composites and that adding more resulted in the composites’ modulus, flexural, and tensile strength decreasing.

Researchers also found that the random distribution of NFR affects the stiffness of composites due to poor stress transfer at the interface during loading. The dynamic behavior of banana–sisal hybrid short fiber-reinforced polyester composites was investigated by Idicula et al. [30]. At 0.40 Vf, they observed a minimum peak height and maximum width for the material loss factor. It was revealed that composites with 0.40 Vf possessed higher stiffness and maximum energy. Further increasing the fiber content in the matrix reduced its stiffness due to the non-uniform fiber distribution. Similar variations were observed by Doan et al. in jute fiber/polypropylene composites [31]. Pothan et al. [32] found that 40 wt% fiber loading enhanced the storage modulus and glass transition temperature of banana fiber composite materials. They also observed that high fiber loading decreased the stiffness of the composites.

Kumar et al. [33] compared the free vibration and damping behavior of short banana and sisal fiber polyester composites. The authors found that banana fiber with a length of 4 mm and sisal fiber with a length of 3 mm at 50 wt% improved the damping and mechanical characteristics. For longer fiber length, the damping properties of composites decreased due to agglomeration. Tayeb [34] investigated the tribological properties of sugarcane fiber–polyester composites and found that the wear rate of composites decreased when fiber length varied from 1 to 5 mm. Further, increasing the length of the fiber resulted in an increased wear rate and friction coefficient of the composite material. Higher fiber length increased the amorphous nature of the composite. Shalwan and Yousif [35] investigated the mechanical and tribological behavior of polymeric composites based on natural fibers. They came to the conclusion that the properties of composite materials are impacted by fiber orientation, fiber length, and volume fraction. Yusuf et al. [36] looked into the tribological characteristics of oil-palm fiber-reinforced polyester composites and discovered that oil palm/polyester composites had better wear properties.

From the results of these reported studies, it is concluded that the properties of natural fibers with short form depend on the fiber aspect ratio, and improvements in properties are generally observed only up to a certain wt%. The main problem associated with short and randomly oriented fibers in composites is achieving uniform distribution in the polymer matrix [37]. Furthermore, it creates a poor interfacing bond between the fiber and the matrix due to a higher weight percentage, resulting in poor mechanical properties.

## 3. Woven Natural Fiber Composite

To overcome the disadvantage of natural fiber with short and random orientation, researchers focused more on the reinforcement effect by incorporating WNFR to enhance the properties of NFCs for low and medium load applications. Because of its ease of processing, low fabrication cost, and improved characteristics, the idea of employing WNFR to produce NFCs was generally adopted. Due to stronger fiber–matrix bonding, the gap between warp and weft acts as a mechanical interlock among the polymer matrix, increasing resistance to failure under load. In addition, the chances of failure are less/delayed due to fiber pullout under dynamic loading conditions. In recent years, tremendous development in the textile sector has motivated researchers to explore the possibilities of improving natural fiber composite properties, making them suitable for many applications. Nowadays, natural fibers are used in continuous and woven forms, which further increases the inherent properties of NFCs. Several researchers analyzed the outcome of weaving patterns such as plain, twill, stain, and basket weaving patterns on the mechanical properties of NFCs. Results revealed that NFCs reinforced with NFR with varied weaving patterns exhibit improved mechanical properties. John et al. [38] and Pothan et al. [39] explained the advantages of various weaving structures such as plain, basket, twill, and satin. Out of these four patterns, plain weave gives uniform distribution, good stability, and porosity. A continuous yarn moves in the warp and weft directions in a regular 1x1 pattern in a plain weave. Plain weave has the major drawback of having a larger crimp in the warp, and the weft impacts the properties of the succeeding composite. To enhance the properties of composite materials, researchers investigated various weaving patterns and evaluated them against plain weave as a reference. Alavudeen et al. [40] tested a woven banana/kenaf polyester composite against a randomly oriented fiber composite with the same wt%. In comparison to the short fiber composite, they discovered that the woven composite had better mechanical characteristics. As a result, it has been demonstrated that continuous natural fiber improves composite performance when compared to composites made with short natural fiber with random distribution.

### 3.1. Mechanical Properties

**Figure 5:**
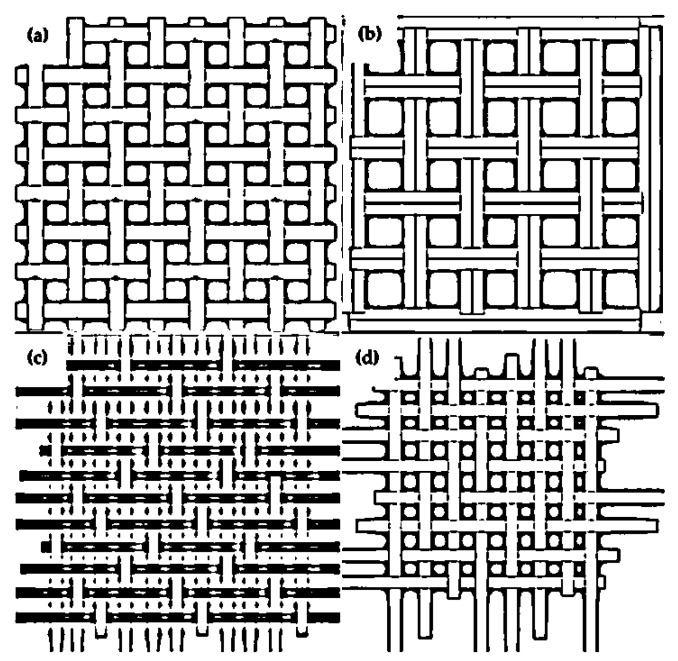
Schematic of various woven mats: (**a**) plain; (**b**) basket; (**c**) twill; (**d**) satin.

The mechanical properties of natural fiber composites, such as impact, flexural, and tensile strength, are influenced by fiber percentage in the matrix, fiber strength, fiber–matrix adhesion, fiber orientation, concentration, and treatment type [41,42]. Steel, titanium, and aluminum were formerly the materials of choice for engineering, civil, aircraft, and automotive applications. WNFR composites, on the other hand, offer favorable weight characteristics and bulk strength, making them a feasible substitute for traditional materials since they have stiffness and superior strength [43,44,45]. Schematic diagrams of basic weaving patterns used in the composite field are illustrated in Figure 5.

In an experimental investigation, Asim et al. [46] evaluated the flexural characteristics and tensile strength of tri-layer palm oil and woven jute fiber–epoxy composites to palm oil–epoxy and woven jute–epoxy composites. Three-layer palm oil and woven jute fiber–epoxy composites had greater mechanical properties than identical composites made with other combinations. It was also discovered that the kind of fiber and its hybridization had an impact on composite characteristics. The mechanical characteristics of Cotton and Kapok fabrics as reinforcing components in a polypropylene matrix were examined by Mwaikambo et al. [47]. They discovered that adding fabric to the composite material enhanced its rigidity. Sapuan et al. [48] studied the mechanical characteristics of woven banana/epoxy composites and discovered that woven banana composites had a higher strength and modulus. The influence of a stacking arrangement on the mechanical properties of sansevieria cylindrical–coconut sheath polyester composites was investigated by Bennet et al. [49]. The maximum modulus was seen when the mat fiber was kept as an exterior layer and short-fiber mat was used as the core material.

Carmisciano et al. [50] investigated the flexural properties of a basalt woven fiber-reinforced vinyl ester composite and a glass fiber composite. Basalt woven fiber composites outperformed glass fiber composites. Venkateshwaran and Elayaperumal [51] investigated the mechanical properties of woven banana–jute–epoxy composites with various stacking sequences. They discovered that adding jute fiber as a core layer increased the flexural and tensile properties of the composite over the jute and banana composites individually. The flexural characteristics of woven pandanus and banana fabric composites with short fiber reinforcement were compared by Mariatti et al. [52]. They discovered that at the same volume %, the woven fabric composite exhibited a high modulus and strength. Finally, Khan et al. [53] investigated the mechanical characteristics of non-woven jute and plain-woven jute composites in the warp direction. They observed that in the warp direction, the woven mat composite outperformed the non-woven composite in terms of mechanical properties.

Rajesh and Pitchaimani [54] analyzed the effect of weaving patterns on mechanical properties compared with composites reinforced with randomly oriented natural fibers. Results revealed that for the same weight percentage, the woven composite improved the mechanical properties of the composites whereas randomly oriented SNFR failed relatively. Short-form reinforcement experienced higher stress concentrations as fiber discontinuity affected the bonding strength between the fibers and the matrix. It led to early failure of the composites compared to woven fabric reinforcement. The individual strength of the yarn and the amount of fiber present in the reinforcement influenced the load-carrying behavior of the composites. Similar observations were made by Alavudeen et al. [40]. They analyzed the effect of fiber strength and weaving patterns on the mechanical properties of polyester composites and compared them with randomly oriented composites. They found that irrespective of fiber strength, the weaving pattern significantly affected the strength of the composites.

Figure 5 depicts commonly used weaving patterns in the composite field, such as plain, basket, twill, and satin weaves. The main advantage associated with plain and basket weaving is the uniform orientation of the fibers in the weft and warp directions. In satin and twill weaves, the fabric will bias diagonally, which influences the load-carrying behavior of the composites. In plain and basket weaves, stress is distributed uniformly along with the warp and weft directions, which affects the mechanical properties of the composites. In twill and satin weaves, stress transfers non-uniformly and diagonally to the warp and weft directions, leading to earlier failure of the composite under loading. In the textile industry, the huckaback style is commonly used in fabrics. Due to the periodic yarn arrangement in both the warp and weft directions, the huckaback pattern enhances the fabric’s surface roughness. The gaps between subsequent strands in the warp and weft orientations are the fundamental drawback of huckaback woven composites, which causes them to break prematurely. As a result, there is a higher concentration of tension during loading. Goutianos et al. [55] studied the effects of yarn twist for woven composites. Results indicated that a higher yarn twist improved the properties of the composites, whereas a lower yarn twist exacerbated insufficient loading capacity. Pothan et al. [56] evaluated the mechanical characteristics of several types of woven sisal fiber composites and discovered that plain-woven fabric improved the composite’s properties. Shibata et al. [57] investigated the flexural strength of bamboo/kenaf fiber-reinforced composites that were unidirectional and randomly oriented. They concluded that the woven fabric, regardless of material, flexural strength, and modulus of the composite, was improved. Table 1 shows the mechanical properties of frequently used plant fibers in the field of composites.

### 3.2. Dynamic Mechanical Properties

Thermal and dynamic mechanical characteristics of newly developed materials are significant parameters to be examined primarily for structural applications. At higher temperatures, the interactions between molecules in materials made out of conventional materials will be higher, which increases energy dissipation and lowers the stiffness. The fiber or yarn arrangement, reinforcement, amount of fiber in the matrix, and adhesion between the matrix in the space between two fiber yarns influence the dynamic characteristics of composite materials. Rajesh and Pitchaimani [83] investigated the dynamic mechanical characteristics of composite materials using weaving patterns and fiber strengths. They discovered that in the glassy zone, regardless of the weaving pattern, the composite had a small change in storage modulus. However, compared to satin, plain, huckaback, and twill woven composites, the basket-design jute composite significantly increased the storage modulus after the glassy area. At higher temperatures, the basket-design composite enhanced structural stiffness and improved resistance to free molecular movement. The basket-woven fabric’s fiber yarn arrangement also reduced stress concentration and supported more weight between two consecutive yarns in the weft and warp directions. Furthermore, the list of published research work that has been conducted to demonstrate the dynamic mechanical properties is tabulated in Table 2.

The impact of weaving patterns on the dynamic mechanical behavior of banana–epoxy composites was investigated by Venkateshwaran and Elayaperumal [51]. The composite enhanced the storage modulus of the composite laminate while having no influence on the glass transition temperature compared to twill and satin weaves. According to the authors, the orientation of natural fiber yarns in the warp and weft directions influenced the storage modulus of the plain-woven composite. In plain weave, a different strand arrangement in the warp and weft orientations enhances stability and minimizes porosity. The high crimp present in both the warp and weft directions is the fundamental issue with plain weave. Plain weave, however, is more rigid than satin or twill. Fangueiro and Rana [99] investigated the viscoelastic behavior of twill and plain-woven hemp fiber-reinforced polylactic acid composites. They discovered that twill weave improved the composites’ viscoelastic and mechanical characteristics, as well as their loss and storage moduli. Gupta [100] discovered that plain-weave reinforcement improved the composite’s dynamic mechanical characteristics more than short fibers. A dynamic mechanical investigation of oil-palm empty fruit bunch (EFB)/woven jute fiber (Jw) epoxy hybrid composites was explored by Jawaid et al. [101]. The woven jute composite’s storage modulus was found to be higher than that of the hybrid composites. It revealed that the hybridization of oil-palm empty fruit bunches with woven jute fabric affects the performance of the composite under the thermal environment due to the addition of oil-palm empty fruit bunches minimizing the resistance of free molecule movement in the polymer chain. Thus, it minimizes the resistance against free molecular movement and reduces stiffness. Asim et al. [102] studied the influence of jute fiber loading on the dynamic mechanical behavior of oil-palm epoxy composites. The inclusion of jute fiber in the oil-palm–epoxy composites increased their storage modulus. It showed that adding high-strength jute fiber to the matrix prevented free molecule movement and improved the composite material’s stiffness at higher temperatures. The dynamic mechanical behavior of PLA–hemp bio-composites was studied by Durante et al. [103]. They discovered that increasing the fiber ratio in the PLA matrix enhanced the composite material’s glass transition temperature and storage modulus. The dynamic mechanical behavior of aliphatic–aromatic co-polyester and green composites consisting of woven flax cloth matrix was studied by Chandrasekar et al. [104]. Conferring to the results, the addition of woven fabric significantly increased the storage modulus of the green composite.

### 3.3. Free Vibration Behaviour

The materials used for structural applications must have superior damping properties, along with strength and stiffness. These properties are significantly influenced by the manufacturing process, type of reinforcement, and matrix. Researchers have fabricated composite laminates using a compression-molding process and compared them with a hand lay-up technique. Results revealed that composites fabricated using the compression-molding technique exhibited improved properties compared to those produced using the hand lay-up method. Kumar et al. [33] reported that the compression-molding process showed enhanced material properties and stiffness, along with energy dissipating properties. For structural applications, it is important to reduce the resonant amplitude of vibration to protect the components and structures from failure. The modal damping associated with each mode of the structure has a considerable impact on the resonant amplitude of vibration. A small exciting force can induce high amplitude vibrations at resonance due to any sizeable vibratory inertia force. In general, fiber-reinforced composites have higher damping properties than conventional materials due to viscoelastic behavior and fiber–matrix interaction.

Free vibration properties such as natural frequency and damping characteristics of fiber-reinforced composites have been analyzed by several researchers using experimental, analytical, and numerical methods. In free vibration analysis, the composite material’s natural frequency and corresponding damping factor were found using the fast Fourier transfer (FFT) algorithm. It changes a time-domain signal to a frequency response signal and provides an incessant peak for the corresponding natural frequency of the composite material. Chandradass et al. [105] experimentally analyzed the outcome of nanoclay additions on free vibration characteristics of a glass fiber-reinforced composite structure. The second-phase nanoscale dispersion in the matrix and E-glass fiber greatly improved the internal damping of the hybrid composites, according to the dynamic results. Gibson [106] analyzed the modal vibration response quantities of composite materials and structures. Results revealed that impulsive excitation methods gave accurate values for the characterization of intrinsic material properties.

Recently, synthetic fibers have been replaced by natural fibers as reinforcements in the polymer matrix because of their better energy-dissipating behavior [107,108]. The development of green composites increases the usage of plant wastes, thereby reducing their carbon footprint. The free vibration behavior of woven reinforced materials improves the natural frequency of the composite material [109,110]. Rouf [111] analyzed the influence of plain, twill, and satin weaving patterns on the dynamic behavior of woven fabric composites. The author found that plain weave increased the damping properties of composites more than satin composites. Duc et al. [112] conducted a modal analysis to determine the natural frequency and damping behavior of unidirectional, laminated, and woven flax fiber (FF)/epoxy composites. They critically evaluated the factors affecting the natural frequency and damping factor of the composite material. They found that the impregnation quality, fiber/matrix adhesion, strength of the fibers, twist of the fiber yarns, and yarn crimp significantly affected the fundamental natural frequency and corresponding damping factor of the composite structure.

Similarly, the effects of structure type, type of fibers, and physical properties such as density, thickness, and manufacturing process on the stiffness of the composite laminate influence the dynamic properties [113]. Mishra and Sahu [114] carried out extensive experimental work on the free vibrational behavior of woven composites with different boundary conditions. They found that the number of layers, fiber orientation, aspect ratio, and different boundary conditions of the woven fiber composite significantly influenced their stiffness values.

According to Chandra et al. [115], fiber-reinforced composites offer better strength and stiffness, as well as a stronger damping effect, than traditional materials. Da et al. [116] measured the frequency and conducted modal damping analysis for jute/sisal hybrid polyester composites using the impulse hammer technique. They found that the average damping factor attained for the jute/sisal hybrid composite was 1.15 times higher than the composite reinforced with the jute layer alone. It was due to differences in the flexural stiffness of the jute/sisal hybrid polyester composite. Rajini et al. [117] discussed the free vibration behavior of coconut woven mat with different percentages of nanoclay added to the polyester composite. The introduction of nanoclay increased the natural frequency of the composite by up to 3 wt%, whereas further addition reduced the matrix stiffness. The damping characteristics of the composite material improved as the wt% of the nanoclay increased, owing to the efficient interaction between the fiber and matrix, which boosted the composite material’s energy dissipation. Rajesh et al. [83,118] reported similar observations for a banana–jute intra-ply hybrid composite. Results showed that the use of a basket-woven composite as reinforcement enhanced the first three fundamental natural frequencies of the composite material. Fiber orientation within the yarn plays an essential role in determining natural frequencies [119,120]. Rajesh and Pitchaimani [121] analyzed the natural frequency of woven natural fiber composites under a buckling load. Results revealed that the weaving patterns influenced the resistance against a buckling load.

## 4. Effect of Hybridization

Hybridization offers substantial improvement in the properties of composites, which has attracted the attention of researchers for structural applications. In general, a significant weight percentage of synthetic fibers such as carbon, glass, etc., are hybridized to enhance the inherent properties of natural fiber composites for the intended applications. For compressive applications, jute fibers are reinforced with glass fiber. However, the hybridization effect is not significant for tensile load applications. Thus, it reveals that the procedure requires applications regarding the hybridization of natural fiber with synthetic fiber to achieve the desired goals/applications. For example, Mansor et al. [122] carried out an experimental investigation, selecting natural fiber and its hybridization for automotive brake lever applications. They found that kenaf fiber with glass fiber provided superior properties for the intended application compared to other natural fibers. The enhancement in the hybridization performance increased the load-carrying behavior of the composites compared to when they were reinforced in the matrix alone. However, the addition of synthetic fiber improved the properties of the natural fiber composite, and the compatibility between the fiber/filler and matrix influenced the failure of the hybrid composites. The list of research work carried out in natural fiber hybrid polymer composites is illustrated in Table 3.

The primary purpose of the hybridization of synthetic fiber/filler in the polymer matrix with natural fiber is to enrich the properties of composites, especially mechanical properties. It is determined by the filler dispersion in the polymer matrix, fiber distribution, fiber adherence to the matrix, fiber contact area, reinforcement high load-bearing behavior, fiber origin, surface treatment (physical or chemical), and stacking sequence. Through experimental studies, most researchers reported that strength enhancement depends on fiber loading, treatment, aspect ratio, and contact area [151,152,153]. Due to hybrid composites’ enhanced strength and land-bearing capacity, they are used in many automotive applications such as car interior components and door panels.

### 4.1. Hybridization of Natural Fiber with Man-Made Fiber

The hybridization of synthetic fiber with natural fiber helps to enrich the properties and to increase the resistance against water absorption and minimize environmental issues and cost. Though natural fibers have many advantages over man-made fibers, many hydroxyl groups present in cellulosic substances such as cellulose, hemi-cellulose, and lignin increase the interaction rate with moisture present in the environment, making them incompatible with hydrophobic applications. Researchers carried out a lot of experimentation on the hybridization of synthetic fiber with natural fiber. They established that the hybridization of synthetic fiber enhanced the mechanical properties of the composite. The improvement is due to the decrease in water absorption and enhancement in bonding. The major problems of natural fibers are moisture absorption due to the interaction of cellulosic substances available in the fiber cell wall. It can restrict the applications of natural fiber composites in various applications, especially outdoor applications, where the interaction of water particles is high. The water absorption of natural fiber composites is affected by the nature of the fiber, growing environment, volume fraction, and diffusivity. Water can enter through available micro-cracks in the matrix and microgaps between the fiber and matrix. Transportation of water particles can happen due to capillary action in the interface gap due to poor wetting. Therefore, composites exposed to the moisture environment can experience debonding and crack formation. In addition, those water molecules act as a plasticizer and reduce the load-carrying capacity, thus minimizing the mechanical properties of natural fiber composites. Henceforth, it is important to improve the resistance to water absorption behavior. Physical and chemical surface modification of natural fiber improves the compatibility of natural fiber with the polymer matrix. However, using a high concentration of harsh chemical treatment affects human health and the environment differently. Hence, to overcome the problem related to the moisture absorption and wettability of natural fibers, the hybridization of synthetic fiber and natural fiber is a better choice. It reduces the water molecule interaction and enhances the properties under various loading conditions for intended applications.

Panthapulakkal and Sain [153] analyzed the impact of the water absorption of hemp fiber composites on tensile strength. They found that the inclusion of glass fiber in the hemp composite increased the tensile properties. In the case of neat hemp composite, debonding arose between the fiber and matrix due to the penetration of water molecules and reduced the strength of the composites. Similarly, the inclusion of glass fiber in the jute composite enhanced the tensile strength of the composite for water-immersed samples. Results revealed that the inclusion of synthetic fiber in the natural fiber composite enhanced durability [154].

Kureemun et al. [155] and Ramprasad [156] found that replacing a small wt% of jute fiber from the polyester matrix with carbon fiber enhanced the mechanical properties of the composites. Due to the high modulus of carbon fiber reinforcement, it compensated for the shortcomings of jute fiber and enhanced its properties. Ridzuan et al. [157] hybridized grass fiber with glass fiber. They found that the hybridization effect enhanced the composites’ storage modulus and increased the composites’ glass transition temperature. The addition of glass fiber in the matrix increased the resistance against free molecule movement in the polymer chain. A similar effect was observed by Romanzini et al. [158], who found that neat resin had a high damping factor, whereas hybridization exhibited a high modulus.

Thwe et al. [159] compared the mechanical properties of short-fiber bamboo composites with hybrid bamboo–glass–polypropylene composites. They reported that the addition of glass fiber up to 20 wt% enhanced the mechanical characteristic of the composite material. Comparable outcomes were observed by Sreekala et al. [160] and Thwe et al. [161] for hybrid phenol–formaldehyde-based glass and oil-palm fibers. Ahmed and Vijayarangan [162] prepared woven jute fiber with woven glass fiber composites to investigate flexural and tensile strength. They found that the properties of the jute composite improved when the glass fiber was maintained as an external layer. The effects of glass and ramie natural fibers on dynamic mechanical behavior were investigated by Romanzini et al. [157]. They discovered that adding glass fiber to the composite material made it stiffer. Ramesh et al. [163] found that incorporating glass fiber into the jute–sisal composite improved the mechanical properties due to minimized internal cracks. It also improved the rigidity and stiffness of the composite. A similar nature was observed by Kumar et al. [164], who found that the addition of glass fiber to bamboo increased the storage modulus. Anuar et al. [165] observed that the hybridization of carbon and kenaf fibers increased the stiffness of the composite material. Simultaneously, it reduced the damping of the composite material, with similar effects observed by other researchers [166,167,168,169,170]. The inclusion of glass fiber in wood polymer composites increased the wear resistance of the composite due to hybridization effects. The impact characteristics of sisal–glass composites were studied by John and Naidu [171]. They discovered that combining sisal and glass fibers improved the composite’s impact resistance compared to a pure sisal fiber composite. Pothan et al. [172] identified that the addition of glass woven fabric in a banana composite enhanced the composite material’s structural properties. The addition of small amounts of glass fiber also increased the modulus and load-carrying capacities of the composites [173,174]. The influence of glass and jute on the woven fabric-stacking sequence on tensile and flexural characteristics was tested. They found that the inclusion of glass woven mat increased the properties of the jute composite. Valente et al. [175] studied the effect of wood flour and glass fiber in the thermoplastic on its flexural properties. They found that the addition of recycled glass fiber in the wood flour thermoplastic composite enhanced the properties of the composite. Amuthakannan et al. [176] found that combining woven basalt and jute fiber composites with varied stacking sequences improved the composites’ deformation. The impact of glass fiber reinforcement on the mechanical characteristics of sisal and jute fiber composites was investigated by Ramesh et al. [177]. They came to the conclusion that adding glass fiber to composite materials improved their properties. Bennet et al. [178] investigated the dynamic properties of coconut sheath–sansevieria cylindrical fiber composite materials. They found that composites with a coconut sheath–sansevieria cylindrica–coconut sheath stacking sequence enhanced the fundamental natural frequency of the composite material. Kumar et al. [179] analyzed the influence of the layering sequence of coconut sheath and banana fiber on the composite. They observed that composites with a higher amount of banana fiber enhanced the mechanical properties. The influence of sequencing on the dynamic mechanical characteristics of plain-woven jute fabric–carbon composites was investigated by Sezgin et al. [180].

Results showed that composites with carbon fiber as the outer skin layer exhibited enhanced dynamic properties and an increased glass transition temperature of 75 °C. Mochane et al. [181] found that 40 wt% sisal/hemp fiber-reinforced hybrid composite gave the best flexural strength compared to hybrid composites with different wt%. Vaghasia and Rachchh [182] found that 9 wt% bamboo fiber in woven bamboo glass–polyester hybrid composite material gave excellent tensile and flexural properties. Flax fiber reinforced with glass fiber was studied for its mechanical properties as a substitute for bone plate fracture fixation. They found that compared to metallic plates, glass/flax/epoxy hybrid composite was significantly more flexible [183]. Sanjay et al. [184] fabricated a hybrid composite using jute/kenaf and a glass layer, and compared the impact properties with a composite prepared with pure jute, kenaf, and glass fiber composites. Results showed that hybridization improved the impact properties of the composite.

### 4.2. Hybridization of Natural Fiber with Natural Fiber

Natural fiber composites with two or more types of natural fiber reinforced in the polymer matrix have better inherent properties and are more compatible than conventional materials for low/medium load applications and automobile applications such as home applications, outer car body, agricultural, interior parts, and packing applications. Edhirej et al. [185] found that natural–natural fiber hybridization alters the tensile properties of the composites. Results revealed that the hybridization of natural fiber with other types of natural fiber composites is a suitable alternative to man-made composites. Das [186] hybridized jute fiber polyester composite with an un-shredded newspaper. They used two stacking sequences, such as jute/newspaper/jute and newspaper/jute/ newspaper. They found that the tensile properties of the hybrid composite were higher than pure newspaper/polyester composite but less than pure jute composite. This revealed that the selection of reinforcement for hybridization depends on the types of fiber and its strength. In addition, it was observed from the result that newspaper reinforcement in the jute composite had a short form and random distribution. Thus, it decreased the modulus of the composites and decreased the load-carrying capacity. Fragassa et al. [187] carried out an experimental investigation on the mechanical properties of flax and basalt fibers and their hybridization. The inclusion of flax fiber reduced the basalt fiber composite’s brittleness, enhanced its plastic behavior, and further postponed the composites’ failure. Dhakal et al. [188] analyzed post-impact mechanical properties of basalt/hemp fiber hybrid composites. Hybrid laminates were subjected to impact load (3, 6, and 9 J) and subjected to quasi-static and cyclic flexural tests. It was found that the addition of basalt fiber enhanced the damage tolerance of the hybrid composite. Kona [189] studied banana fiber-reinforced composite mechanical properties using groundnut shell ash as filler. Results revealed that the accumulation of filler enhanced the mechanical properties of the composites, as the addition of filler enhanced the adhesion between the banana fiber and the matrix. The reinforcement of the filler along with the fiber increased the resistance against the crack formation and microvoids. Thus, it enhanced the properties of the composites. Sarasini et al. [190] used ramie and borassus fibers and developed biodegradable composites using polycaprolactone. Results revealed that the ramie fiber composite increased the brittle nature of the composites, whereas borassus enhanced the elasticity of the composites. The inclusion of both fibers in the matrix raised the tensile strength and crystallinity of the composites, demonstrating improved performance. Jumaidin et al. [191] created a hybrid composite using seaweed and sugar-palm fibers with starch as a matrix and discovered that hybridization improved the composites’ tensile characteristics compared to seaweed and sugar-palm-reinforced composites.

Though hybridization addresses the drawbacks of single-fiber reinforcement, the incompatibility of hybrid reinforcement in the hydrophobic matrix is exacerbated by the presence of additional hydroxyl groups in the cellulose and hemicellulose in the fiber cell wall. As a result, the benefits of natural–natural fiber-reinforcing hybridization are diminished. As a consequence, increasing the interfacial bonding strength of hybrid reinforcement and matrix is essential. For example, when two stronger natural fibers are reinforced (jute/flax, jute/hemp, sisal/jute, etc.) in the hydrophobic matrix, it increases the interaction with water molecules present in the environment. The reason behind higher interaction with water molecules associated with stronger natural fibers is that the presence of more cellulose contributes to interaction with moisture. Thus, it reduces the interfacial adhesive bonding. Major parts of cellulose have a significant –OH-to-C ratio, which interacts with water molecules. Cellulose is semi-crystalline, and the crystalline region provides a barrier against water penetration, whereas the amorphous region of the cellulose allows the water molecules to pass through. Therefore, it affects the cellulosic chain and losses its stiffness. Hence, it minimizes the advantages of natural–natural fiber hybridization by increasing the incompatibility between fiber and matrix, generating more microvoids and delamination. King et al. [192] analyzed the influence of bagasse/basalt hybrid reinforcement of polylactic acid (PLA) composites on tensile, flexural, and water absorption behavior. They found that as the amount of fiber increased in the matrix, the weight loss rate increased. This is due to the interaction of moisture increasing the degradation rate of the composites. Gupta and Srivastava [193] analyzed the effect of sisal/jute fiber hybridization on mechanical properties and water absorption behavior. They found the maximum water uptake, sorption, diffusion, and permeability coefficient. Almansour et al. [194] investigated the effect of water absorption on the fracture toughness of flax/basalt-reinforced vinyl ester hybrid composites.

Researchers modified the fiber surface using physical and chemical methods to improve the adhesive strength between fiber and matrix. In the chemical modification, alkali treatment is commonly used by researchers and has been noticed to improve the adhesion between fiber and matrix by removing a substantial amount of hemicellulose and lignin from the fiber cell wall. Kabir et al. [195] used alkali, acetyl, and silane chemical treatments and separated cellulose, hemicellulose, and lignin from the hemp fiber. TGA results revealed that hemicellulose degraded faster than cellulose and lignin. They found that the hydrophilic nature of hemp fiber was reduced by treating it with a higher concentration of sodium hydroxide (NaOH), followed by acetylation, whereas silane treatment acted as a coupling agent. The fiber cell wall of Napier fiber collapsed by treating it with 2% and 5% alkali solution [196]. Cao et al. [197] treated bagasse fiber with alkaline solution and found that a lower concentration enhanced the mechanical properties. The bonding strength between the fiber and matrix was further enhanced by changes made to the fiber surface, such as raising the aspect ratio and improving mechanical interlock. Gassan and Bledzki [198] investigated jute/epoxy composite mechanical properties using alkaline treatment. Alkaline treatment enhanced the fiber’s stiffness, which helped to increase the inherent properties of the composite. Other than alkali chemical treatment, silane, acetylation, benzoylation, acrylation, maleated coupling agents, isocyanates, and permanganate have also been used by researchers and have enhanced the properties of composites [199]. Zaman et al. [200] investigated the effects of urea and potassium permanganate on the mechanical characteristics of jute/polypropylene composites. A lower concentration of urea and potassium permanganate improved the mechanical characteristics of the composites, whereas higher concentrations reduced the properties of the composites due to the collapse of the cellulosic structure of the fibers. Morphology analysis revealed that the fibers became thin and rough due to removing a certain quantity of hemicellulose and lignin. NaOCl, NaOCl/NaOH, and H_2_O_2_ solution removed the hemicellulose and lignin from green coconut fiber. SEM results revealed that H_2_O_2_ removed a high amount of the waxy and fatty acid but did not alter the surface.

In contrast, NaOCl/NaOH minimized the hemicellulose content from the fiber wall. NaOCl treatment did not affect it because it increased the hydrophilic nature of the fiber [200,201]. The storage and loss moduli of jute fiber-reinforced composite was enhanced using an alkaline treatment by Ray et al. [202]. The treatment enhanced the glass transition temperature of the composites. Similarly, researchers adopted physical modification using coating technology to enhance the adhesive strength of the fiber reinforcement in the matrix. Costa et al. [203] used a graphene oxide coating to prepare acuraua fiber-reinforced epoxy composite. Results revealed that surface coating enhanced the adhesion. Kan [204] used plasma treatment and modified the surface properties of textile material. They found that plasma treatment introduced a functional effect. Vellguth et al. [205] enhanced the thermal stability of a natural fiber composite through an epoxy coating on the natural fiber using the spray-coating method. Esen et al. [206] used thermal vacuum evaporation and coated Al and Zn on textile material. Both the coatings improved the electromagnetic interference and UV/IR (ultraviolet/infrared)-shielding properties. Jute fibers were treated with cold argon plasma for 5, 10, and 15 min and reinforced in the polyester matrix. Plasma treatment altered the fiber from hydrophilic to hydrophobic. It enhanced the fiber/matrix adhesion [207].

### 4.3. Hybridization of Natural Fiber with Filler

A higher contact area associated with filler material helps to establish better bonding between natural fiber reinforcement and polymer matrix, leading to improved mechanical properties by increasing the resistance against debonding and the initiation of cracks and the random propagation of them, and increasing the rigidity of the composites. However, reinforcement by fillers in polymer composites sometimes decreases the composites’ strength, stiffness, and modulus. This is due to the depression of the fillers in the polymer matrix, thus affecting the properties of the composites [208]. In general, oil-palm nanofiller, montmorillonite (MMT), organically modified montmorillonite, fly ash, graphine, carbon nanotube, coir nanofiller, carbon black and cellulosic nanofiller, and rice husk ash fillers are reinforced along with natural fiber reinforcement in the polymer matrix, which leads to enriching the properties of composites [209]. Arrakhiz et al. [210] observed that the weight percentage of clay in the polymer composite enhanced the properties. However, a higher weight percentage showed no improvement in properties. They reinforced clay filler along with pinecone fibers. Results revealed that the addition of clay filler enhanced the properties of the composite for 30 wt% loading, whereas for a higher wt% filler did not show a significant contribution to strength improvement. Njuguna et al. [211] compared the influence of filler diameter on strength and the stiffness of various polymers such as polyesters, polyamides (PAs), polypropylenes (pps), polyurethanes (PUs), and high-performance/temperature engineering polymers such as polyarylacetylene (PAA), polyimide (PI), poly (ether ether ketone) (PEEK), and polyphenylenee benzobisoxazole (PBO). They found that when the particles shrank from macro to sub-micro and nano-level, it led to superior mechanical properties due to the large surface area-to-volume ratio of the fillers. Saba et al. [212] investigated the influence of nano oil-palm empty fruit bunch reinforcement on the dynamic mechanical behavior of the composites. They found that the addition of nano oil-palm empty fruit bunch enhanced the modulus value of the composite, and 3 wt% altered the damping factor. Thus, it increased the usage of structural applications because it required both strength- and energy-dissipating behavior.

Similarly, the tensile properties of madar fiber were enhanced by adding nanoclay to the polyester matrix [213]. The decrement in the mechanical properties of the composite with high filler loading is because the random dispersion of the higher filler loading in the polymers increased the agglomeration. Thus, it increased the incompatibility nature of the enhancement of its strength and stiffness. This is understood from the study carried out by Ravichandran et al. [214]. They reinforced the halloysite nanotube in the epoxy polymer and found that compared to neat epoxy composite, the accumulation of filler decreased the properties of the composites. The reason was the lack of interfacial bonding due to the uniform dispersion of particles. Furthermore, they developed techniques for particle dispersion and found that an ultrasonic homogenized nanocomposite improved the properties of the composites compared to other dispersion methods. Thus, it reveals that the quality of the filler and its dispersion in the polymer influenced the strength and stiffness enhancement of the composites. In addition, it increased the resistance against the failure of the composite by minimizing the initiation of cracks and the random propagation of them in the matrix. Thus, it helped to transfers the stress uniformly from the matrix to the particle under loading.

### 4.4. Intra-Ply Hybridization

**Figure 6:**
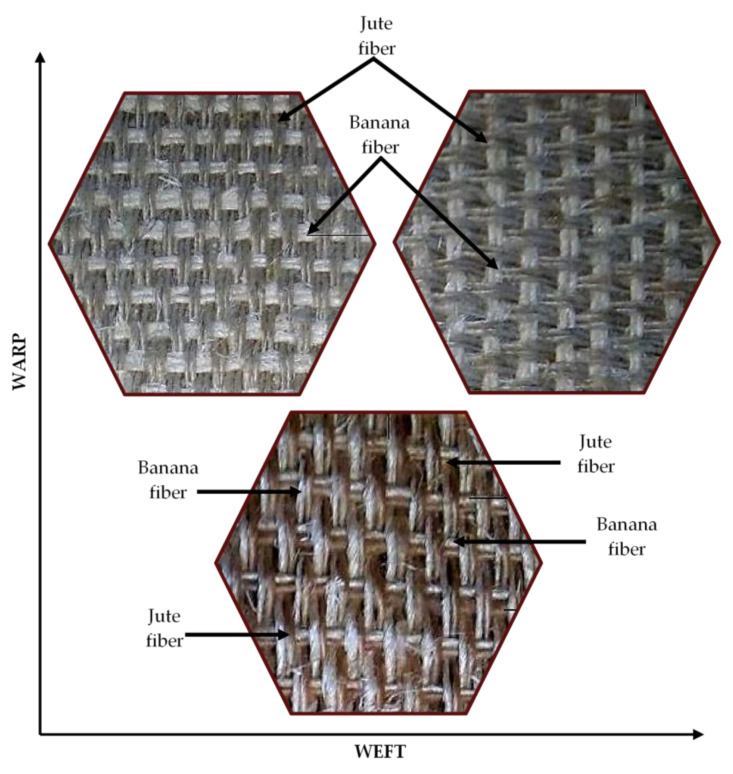
Various intra-ply hybrid woven natural fiber mats.

**Figure 7:**
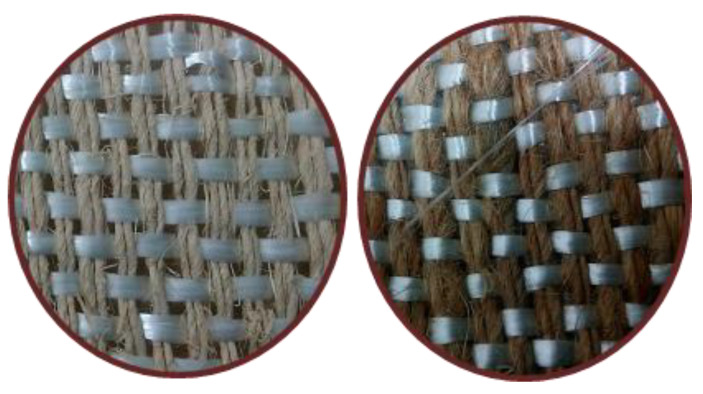
Different types of intra-ply hybrid woven natural/glass fiber mats.

Fiber yarn orientation influences the excellent properties of natural fiber composite. The relatively strong natural fiber yarn orientation in the loading direction exhibits a higher modulus and stiffness in natural fiber composites. In general, natural fiber composites are reinforced in a single-matrix system using natural fiber, synthetic fiber, or filler material to improve their properties. Many researchers investigated the hybridization of natural fiber with synthetic fiber and cellulosic fiber with natural fiber in a polymer matrix in an experimental investigation. They found that hybridization enhanced the mechanical properties of the natural fiber composites compared to natural fiber-reinforced composites alone in the matrix [215,216]. Inter- and intra-ply hybridization is a technique for improving the characteristics of natural fiber composites. Two or more natural fiber yarns are aligned in the warp and weft directions in the hybridization process, avoiding the stacking sequence and fiber orientation of natural fibers in the matrix. Maslinda et al. [217] reinforced the epoxy matrix with interwoven kenaf/jute and kenaf/hemp fabrics and investigated the effect of fiber yarns on the mechanical parameters of the composites. They discovered that hybridization interplay improved the composite’s tensile characteristics due to the presence of more muscular fibers such as kenaf, hemp, and jute in the loading direction, which increased the load-carrying capacity compared to when those fibers were reinforced individually in the matrix. Another reason is that the presence of different yarn fibers in the single woven fabric provided more resistance against fiber yarn failure and compensated for the effect. Figure 6 and Figure 7 shows the various intra-ply hybrid woven mats (natural–natural, natural–glass) [83,181].

Park and Jang [218] fabricated intra-ply fabrics using aramid and polyethylene fiber yarn and carried out an experimental investigation on flexural and interlaminar shear strength (ILSS). They found that compared to intra-ply hybridization, aramid fiber oriented in the loading direction enhanced the flexural strength, and intra-ply hybridization exhibited a lower value of ILSS. This was due to the difference in the failure modes in the flexural and ILSS tests. This revealed that a vast difference in the properties of the fiber yarns present in the woven fabric’s warp and weft directions reduced the performance but increased the resistance against failure. In contrast, the tensile and impact characteristics of E-glass fabric reinforced composite were enhanced by hybridization of polyvinyl alcohol (PVA) polyester fiber in the fabric. They found that the presence of the PVA fiber in the intra-ply fabric enhanced the specific impact energy of the laminates and increased their resistance to crack propagation [219]. Attia et al. [220] compared the intra- and inter-ply hybrid composition mechanical properties of the composites. Results revealed that the presence of the E-glass and polypropylene improved the intra-ply hybrid composite and enhanced the resistance against tensile and flexural loading more than the inter-ply composite. Dehkordi et al. [221] enhanced the impact behavior of basalt and nylon composite using interplay hybridization. The results showed that impact energy, hybridization, and variation in basalt/nylon fiber content all had an effect on the impact performance of composite plates. The dynamic properties of the jute and banana natural fiber composite were enhanced through intra-ply reinforcement [83]. They analyzed the factors affecting the composites’ modulus and energy-dissipating properties, such as fiber strength, fiber orientation, weaving pattern, and several layers. They found that the relatively stronger jute fiber in the loading direction with braided weaving architecture enhanced the storage modulus.

In contrast, the banana fiber in the loading direction enriched the energy-dissipating properties. However, the combination of jute and banana in the single-woven fabric enhanced both the modulus and the damping factor of the intra-ply hybrid composites. The review found that the selection of fiber and its orientation with the exemplary weaving architecture helped improve the properties of the natural fiber composite through intra and inter-ply hybridization.

## 5. Braided Reinforcement

Inherent properties of composite material can be enhanced by reinforcing stiff fiber in the polymer matrix. In natural fiber composites, the stiffness of the natural fiber can be enhanced by replacing short natural fibers extracted from the natural source with continuous fiber yarn, which can be substituted into woven form. Though the woven form enhances the stiffness and modulus of the reinforcement, weaving architecture and interlacing between fiber yarn in the warp and weft directions influences the strength and modulus of the composite. Furthermore, to improve the properties of the composites, researchers have used advanced techniques such as braiding and knitting for natural fiber reinforcement. Compared to conventional yarn, braided yarn enhances the modulus value, enhancing the load-carrying behavior of the yarn. Reinforcement of those braided yarns increases the composites’ inherent properties compared to those reinforced using conventional twisted yarn [221]. Furthermore, it can be used as woven fabric, which can improve the properties of the composites, which is incomparable to natural fibers reinforced in any other form. The properties of braided fabrics depend on the braiding angle. A lower braiding angle enhances the strength and modulus value more than a higher braiding angle [222]. Composites reinforced with braided woven fabric are used in a variety of engineering applications, including structural parts, air ducts, and motor car and household applications. In a study comparing the mechanical qualities of braided, woven, and knitted fabric-reinforced natural fiber composites, it was observed that braided woven jute fabric composites had better mechanical characterization than conventional and knitted woven fabric reinforcement. The fabric Young’s modulus was improved by braided-form natural fiber reinforcement, which also improved load resistance. For confirmation, Rajesh and Pitchaimani [54] carried out an experimental investigation on the tensile strength of braided and conventional yarns and their fabrics. Results revealed that the braided woven fabric and its yarn had a higher Young’s modulus than the other types, which helped enhance the composite laminate’s overall performance. The discoveries showed that a banana–jute intra-ply composite was compared to separate banana and jute woven textiles. Individual jute braided fabric increased the mechanical parameters of the braided composite compared to the banana–jute intra-ply composite. In contrast to the normal woven fabric, the intra-ply banana–jute woven fabric increased the mechanical properties of the composite material. They concluded that the kind of yarn had a higher influence on the mechanical properties of composite materials than the fiber strength. The amount of fiber and the degree of twist defined the braided yarn’s strength. Rajesh and Pitchaimani [83] investigated the dynamic mechanical and free vibration properties of composites experimentally. They observed that jute-braided woven fabric improved the composite material’s storage modulus, whereas knitted jute increased the composite material’s loss factor. Because there is a high level of contact between the fibers and the matrix in knitted fabric, the stress concentration between the fibers in the fabric increases, thus, in turn, increasing the energy-dissipating behavior of knitted composites, irrespective of the fiber or yarn. In addition, free vibration studies on braided composites revealed similar variations. The braided composite showed enhanced fundamental natural frequencies irrespective of fiber or yarn arrangement, whereas knitted fabric composites enhanced the damping factors. From these results, it was concluded that composites with woven fabrics and braided yarn enhanced the properties of the composite [54,119]. The potential of braided composites was discussed by Ayranci et al. [222]. They determined that braided composites may be used in a variety of applications without losing stiffness, stability, or strength and that they are an excellent alternative to conventional materials in structural rods, columns, shafts, pressure vessels, and plates. Sun and Qiao [223] conducted experimental and theoretical studies on the mechanical characteristics of braided composites and discovered that the braiding angle affected the composites’ mechanical properties. The volume average compliance approach was utilized by Sun and Sun [224] to assess the mechanical characteristics of the 3D braided composite. They found that, compared to straight yarn-woven fabric, braided-woven fabric greatly improved the mechanical characteristics. The parameters affecting the mechanical characteristics of braided composites were investigated by Goyal et al. [225]. They observed that the mechanical properties were controlled by the braid angle, waviness ratio, and cross-sectional form. Ye et al. [226] investigated the impact characteristics of a sandwich composite in an experimental setting and found that a sandwich composite with a stiff woven fabric as a face layer and two weak knitted core layers improved the properties. The performance of knitted composites for diverse applications was examined by Leong et al. [227]. Muralidhar et al. [228] compared plain-woven composites to knitted fabric composites in terms of flexural properties. The composite with knitted-woven fabric in the outer layer had higher mechanical characteristics, according to the results. Hu et al. [229] discovered similar patterns. They discovered that the 3D knitted basalt increased the composite material’s Young’s modulus and gave better deformation resistance.

## 6. Knitted Reinforcement

Knitted reinforcement is a special kind of reinforcement that helps overcome the delinquents associated with natural fiber reinforced in short form. It provides good stability and properties compared to unidirectional reinforcement. Woven-fabric reinforcement provides good stability and balances properties in the warp and weft directions due to the high crimp in the warp and weft directions’ tensile modulus of the composites [230,231]. A loop structure associated with knitted fabric tends to deform considerably, but it reduces the mechanical properties of the composites. The development of multi-axial knitted fabric is a good alternative to conventional reinforcement [232]. It can improve the composites’ dynamic properties, as fiber yarns are not crimped [233]. Xue and Hu [234] developed a modified flat knitting machine and fabricated a biaxial weft-knitted structure. The knitted structure lowered the mechanical characteristics of the composites, according to the findings. Muralidhar [235] improved the mechanical characteristics of the composites by using flax plain-woven, rib-knitted fabric. The authors stacked rib-knitted/plain-woven fabric in the epoxy matrix. Results showed that composite with knitted fabric in the outer layer enhanced the flexural and impact properties of the composites. Kannan et al. [236] developed double-faced plain and rib-knitted fabric and found that parallel lamination enhanced the flexural and impact strength of the composites. Carvalho et al. [237] compared the mechanical behavior of plain-woven fabric and knitted fabric-reinforced composites. They observed that woven-reinforced composites outperformed plain weft-knitted fabric composites in terms of mechanical characteristics. Both woven and knitted fabric-reinforced composites had a higher impact strength than plain composites.

## 7. Conclusions and Future Scope

- The influence of reinforcement on the strength improvement of natural fiber composites was thoroughly examined in this report. Short natural fiber reinforcement with random orientations provides poor resistance under loading due to high-stress concentration and rapid crack propagation. Thus, it increases the water-absorbing behavior and reduces the composites’ mechanical properties due to the significant surface contact of cellulosic natural fiber with moisture available in the environment.
- On the other hand, woven reinforcement enhances the tensile, flexural, and impact strength of natural fiber composite due to the presence of natural fiber in continuous yarn form. Thus, it increases the rigidity of the reinforcement and enriches the properties of the composite. However, it reduces the modulus value of the composites due to the high crimp in the fiber yarn in the warp and weft directions. In addition, various chemical and physical surface treatments were reviewed, and it was shown that they increase the interfacial bonding between fiber and matrix. Further, the hybridization of natural fiber with synthetic fiber, cellulosic fiber, and various fillers was reviewed.
- Furthermore, sophisticated weaving techniques such as braiding and knitting were reviewed, with the conclusion that braided composites significantly improve the characteristics of the composite material.
- Knitted composites, on the other hand, improve the energy dissipation capabilities of the composite materials, implying that braided fabric composites could be used in biological and structural applications.
- In future, natural fiber-reinforced composites should be further studied for their fatigue properties and biocompatible properties for possible considerations in biomedical fields such as orthopedic bone plates, total hip replacement, and prosthesis applications.

## Figures and Tables

**Table 1:** Mechanical properties of plant fiber-reinforced polymeric biocomposites.

Composites	Flexural Strength (MPa)	Flexural Modulus (GPa)	Tensile Strength (MPa)	Tensile Modulus (GPa)	Elongation at Break (%)	Author and Year	Ref.
Jute/polypropylene	77.32	4.34	56.71	1.82	–	Chandekar et al. (2020)	[58]
ramie (5-layer) /epoxy	98.73 ± 5.98	–	99.04 ± 2.85	–	–	Darshan and Suresha (2021)	[59]
Kenaf/polypropylene	45.56	2.37	24.67	2.35	–	Akthar et al. (2016)	[60]
Sisal/epoxy	252.39 ± 12.11	11.32 ± 1.02	83.96 ± 6.94	1.58 ± 0.08	–	Gupta and Srivastava (2016)	[61]
Rice straw/LDPE	33.7	1.6	13.7	0.144	24.10	Xia et al. (2018)	[62]
Pineapple/epoxy	~100	–	80.12 ± 2.23	8.15 ± 0.23	–	Odusote and Oyewo (2016)	[63]
Rice straw/polypropylene	36.5 ± 0.5	1.28 ± 0.027	33.2 ± 0.5	1.66 ± 0.025	23.9 ± 2.9	Hidalgo-Salazar and Salinas (2019)	[64]
Reed/citric acid	12.51	2.45	–	–	0.54	Ferrandez-Garcia et al. (2019)	[65]
Basalt fiber/silk fiber/epoxy	151.42	6.20	118.85	2.15	–	Georgiopoulos et al. (2016)	[66]
Sisal/cotton/polyester	270 ± 4	12.62 ± 0.41	65 ± 5	0.52 ± 0.015	12.31	Sathishkumar et al. (2017)	[67]
Hemp/sisal/epoxy	44.47 ± 2	1.892 ± 0.061	31.76 ± 0.88	1.173 ± 0.32	3.2 6 ± 0.41	Thiagamani et al. (2019)	[68]
Sisal/chitosan/epoxy	136 ± 2.8	7.023 ± 0.61	46.70 ± 3.5	3.821 ± 0.13	2.176 ±0.82	Soundhar et al. (2019)	[69]
Sisal/bagasse/epoxy	0.76	–	27.36	–	0.06	James et al. (2020)	[70]
Jute/hemp/flax/epoxy	66 ± 4	1.25 ± 0.23	60 ± 3	1.88 ± 0.21	5.8 ± 2.2	Chaudhary et al. (2018)	[71]
Banana/ramie/polypropylene	30		35 ± 2			Sai krishnan et al. (2020)	[72]
sisal/banana/coir/epoxy	48.60	3.45	26.35	1.20	–	Balaji et al. (2019)	[73]
Date palm/flax/thermoplastic starch	73.6	5	31	2.8	5.25	Ibrahim et al. (2014)	[74]
Kenaf fiber/phenolic resin	62.12	2.63	15.8	4.350	2.89	Naresh Kumar et al. (2021)	[75]
Banana/jute fiber/vinylester	70	3.26	17.98	1.89	4.5	Ravindran et al. (2021)	[76]
Red banana/ramie/vinyl ester	80	–	42	–	–	Sai krishnan et al. (2020)	[77]
Flax/jute/polypropylene	58.79 ± 1.73	1.39 ± 0.11	39.48 ± 1.61	2.85 ± 0.12	2.90 ± 0.18	Karaduman et al. (2015)	[78]
Coconut sheath/epoxy	76.80	–	58.60	–	–	Suresh Kumar et al. (2014)	[79]
Areca sheath/palm leaf sheath fiber/epoxy	51	–	46	–	0.18	Ganesh et al. (2020)	[80]
Kenaf/jute fiber	57.2	4.62	43.21	3.60	2.1	Khan et al. (2019)	[81]
Banana/kenaf/epoxy	24	2.32	54	0.291	18.5	Sathish et al. (2017)	[82]

**Table 2:** Some of the research work related to dynamic mechanical properties.

No.	Composites	Observations	Authors and Year	Ref.
1.	Kenaf and hemp bast fiber-reinforced polyester	The composites had a relatively higher storage modulus than other samples.	Aziz and Ansell (2004)	[84]
2.	Natural fiber-reinforced polyethylene	The developed composite had relatively better shear properties than other samples.	Franco and Valadez (2005)	[85]
3.	Coir fiber-reinforced natural rubber	Interfacial bonding influence energy dissipation was observed.	Geethamma et al. (2005)	[86]
4.	Jute fiber-reinforced green composites	The developed composites had relatively better tensile property and toughness.	Hossain et al. (2011)	[87]
5.	Doum fiber-reinforced polypropylene composites	The usage of a coupling agent in the composites improved the rheological properties.	Essabir et al. (2013)	[88]
6.	Flax- and linen-fabric-reinforced epoxy	Improved fiber/matrix adhesion reduced the damping ratio of the composite.	Yan (2012)	[89]
7.	Coconut sheath fiber epoxy	The enhanced interface bonding reduced the damping ratio of the fiber.	Kumar et al. (2014)	[90]
8.	Banana fiber-reinforced phenol formaldehyde resole	The developed composite had a better glass transition temperature and storage modulus.	Indira et al. (2014)	[91]
9.	Woven coconut sheath/polyester composite	The developed composites demonstrated better damping characteristics than the counterpart materials.	Rajini et al. (2013a)	[92]
10.	Banana/polyester hybrid composites	Reducing the red-mud particle composition increased the damping properties of the composites.	Uthayakumar et al. (2014)	[93]
11.	Ensete stem fibers/polyester composites	The storage modulus of the constructed composites made from ensete fibers treated with 5.0% NaOH was 1412 MPa, i.e., it was 108% more than that of untreated ensete-fiber polyester composites.	Negawo et al. (2019)	[94]
12.	Date palm fibers/epoxy composites	The storage modulus and loss modulus were improved by including date palm fibers (DPF) in epoxy. However, 50% DPF loading showed greater performance than 40% or 60% DPF loading.	Gheith et al. (2019)	[95]
13.	Banana fiber (BF)/recycled high-density polyethylene composites (RHDPEs)	The modulus of the RHDPE matrix was significantly increased when BF was added. An increase in the storage modulus value of about 20.42% was found while adding BF to RHDPE.	Sukanya and Kothapalli (2018)	[96]
14.	Pineapple leaf fiber (PALF) hybridized with basalt-reinforced epoxy composite	Changes in fiber orientations were discovered to have a significant impact on the loss tangent and storage modulus.	Doddi et al. (2020)	[97]
15.	Luffa cylindrical/ polyester composite	The effects of fiber surface treatment (with NaOH, silane, and Ca(OH)_2_) and fiber content on the generated vegetable fiber (luffa cylindrica) polyester composite were investigated (30%, 40%, and 50%). The Ca(OH)_2_-treated fiber had a high peak in the damping factor (at 50%), whereas silane-treated fiber had a higher loss modulus (at 50%).	Kalusuraman et al. (2020)	[98]

**Table 3:** Various studies on natural fiber hybrid polymer composites.

	Hybrid	Matrix	Observations	References
**Natural Fiber and Natural Fiber**	Rice husk/sisal	Polyurethane	A total of 82/18 (% *w*/*w*) rice husk/sugarcane bagasse combinations showed higher mechanical properties.	Otto et al. (2017) [123]
	Bamboo fiber/sisal	Polyester	Tensile strength increased by 30%, flexural strength increased by 27.4%, and impact strength increased by 36.9%.	Prasanna et al. (2016) [124]
	Jute/hemp/flax fiber	Epoxy	The developed hybrid composite exhibited a higher modulus, tensile strength, and impact strength.	Chaudhary et al. (2018b) [125]
	Jute/ramie	Epoxy	Mechanical testing revealed that increasing the quantity of bidirectional woven ramie fiber enhanced the flexural and tensile strength of the hybrid composites, whereas increasing the content of chopped jute fiber lowered the flexural and tensile strength.	Mohanvel et al. (2021) [126]
	Sugarcane bagasse/bamboo	Polyurethane foam	In comparison to other combinations, the bagasse fiber/bamboo charcoal 30/70-based composites had a greater flexural strength, impact strength, and thermal insulation coefficient.	Abedom et al. (2021) [127]
	Caryota/sisal	Epoxy	Over single-fiber composites, hybrid composites exhibited improved mechanical characteristics.	Atmakuri et al. (2021) [128]
	Ramie/sisal/curaua	Epoxy	Hybridization of sisal-based composites improved mechanical characteristics. The thermal investigation revealed that the hybridization had no effect on the composite’s thermal stability.	Pereira et al. (2020) [129]
	Banana/coconut sheath fiber	Polyester	The mechanical properties were varied with the layering sequence of banana and coconut sheath fiber. Irrespective of the relative wt% of the fibers and layering sequence used, alkali treatment exhibited a positive effect on the assessed properties.	Senthil Kumar et al. (2016) [130]
**Natural Fiber and synthetic fiber**	Kenaf fiber/Kevlar fiber	Epoxy	The hybridization of kenaf with Kevlar fiber improved the mechanical characteristics of epoxy composites.	Ramasamy et al. (2021) [131]
	Flax fiber/basalt	Green vinyl ester	The hybrid composite was prepared by using flax fiber reinforcement (FFR) in the central zone and basalt fiber reinforcement (BFR) in the external layers for applications of boats and yachts. The results showed significant impact behavior improvements for hybrid composites compared to single composites.	Zivkovic et al. (2017) [132]
	Sisal fiber/glass	Epoxy	Higher mechanical properties were observed while placing glass fiber as an external layer and sisal fiber as an inner layer.	Soundhar et al. (2020) [133]
	Flax fiber/carbon	Epoxy	Hybrid composites were prepared by using flax and carbon fiber with different stacking sequences. Results revealed that the presence of carbon fiber laminates as outer layers and flax as inner layers showed higher mechanical properties in contrast to other combinations.	Sarasini et al. (2016) [134]
	Basalt/glass fiber	Unsaturated polyester	In comparison to clean glass fiber composites, adding basalt to a glass fiber-reinforced unsaturated polyester resin enhanced the tensile, density, and flexural characteristics of the composites.	Sapuan et al. (2020) [135]
	Bamboo/glass fiber	Polypropylene	The hybrid composites (bamboo–glass fiber) performed minimum heat reduction, and were thermally steadier before starting to degrade at 275 °C and fully degraded at 400 °C compared to glass-polypropylene composites.	Zuhudi et al. (2016) [136]
	Sugar palm fiber/carbon	Epoxy	The ratio of 60/40 hybrid sugar palm yarn/carbon fiber-reinforced composites delivered the best flexural and torsion performances.	Baihaqi et al. (2021) [137]
	Areca sheath/jute/glass	Epoxy	The hybrid composites using jute fiber as middle layers, areca sheath fiber as an inner layer, and glass textiles as an exterior layer showed a significant increase in mechanical properties.	Jothibasu et al. (2018) [138]
	Basalt fiber/Kevlar	Polypropylene	Results indicate that there was a considerable enhancement in the energy-absorbing capability of hybrid composites (Kevlar/basalt/polypropylene) compared to Kevlar/polypropylene and basalt/polypropylene composites.	Bandaru et al. (2016) [139]
	Kenaf fiber/Kevlar	Epoxy	Due to the sandwich structural effect, the hybrid composites had better mechanical characteristics in tension than compression.	Salman et al. (2016) [140]
**Natural Fiber and filler**	Waste cotton/wood sawdust	Polypropylene	The hybrid composites showed higher tensile strength and flexural strength up to 15 wt% of addition of wood sawdust particles in the polypropylene composites.	Islam et al. (2019) [141]
	Prosopis juliflora fiber/CaCO3,/TiO2 and Al2O3	Epoxy	Hybrid composites were prepared by using prosopis juliflora fiber with three different filler materials (CaCO3, TiO2, and Al2O3). The composites with Al_2_O_3_ filler material attained higher mechanical properties than the other two filler materials.	Venkateshwar et al. (2019) [142]
	Kenaf/magnesium hydroxide	Epoxy	The hybrid composites were prepared by the addition of magnesium hydroxide (MH) filler-reinforced kenaf/epoxy hybrid composites with various weight percentages (10%, 15%, 20%, and 25%). When compared to the rest of the hybrid composites produced in this investigation, the 20% MH/kenaf/epoxy hybrid composites had better mechanical strength, thermal stability, and dynamic characteristics.	Saba et al. (2019) [143]
	Bamboo fiber/fly ash	Polypropylene	Hybrid composites were prepared by using bamboo fiber and polypropylene along with different concentrations of fly ash. With the addition of 25 wt% of fiber in the composition, the flexural strength and bending moment were increased.	Venkateswara Rao et al. (2019) [144]
	Coir fiber/graphene nanosheet	Polyester	The mechanical characteristics of hybrid composites with graphene loadings of 1.5 wt% were better.	Abdellaoui et al. (2019) [145]
	Banana fiber/fly ash	Epoxy	The composites were prepared with banana fiber/epoxy and banana fiber/fly ash/epoxy hybrid composites. In comparison to epoxy composite, it was found that fly ash/epoxy composite had better properties.	Kauser et al. (2019) [146]
	Hemp/sisal/silica nanoparticles	Epoxy	Compression molding was used to make hemp–sisal natural fiber-reinforced hybrid epoxy composites with different proportions of silica nanoparticles (0, 1, 2, 3, and 4 wt%). The composites containing 2 wt% silica nanoparticles exhibited maximum tensile strength, impact strength, and hardness.	Singh et al. (2021) [147]
	Sisal fiber/mustard cake/pine needle	Polyester	The hybrid polymer composites based on 40 wt% sisal and 5 wt% pine needles delivered superior mechanical and wear properties compared to other combinations.	Kumar et al. (2017) [148]
	Coir fiber/aramid fiber/coconut shell powder	Vinyl ester	The hybrid polymer composites based on 20 wt% coir fiber, 10 wt% aramid fiber, and 5 wt% coconut shell particles showed a 52% increase in hardness, 145% increase in tensile strength, and 75% increase in the modulus compared to other combinations.	Udaya Kumar et al. (2018) [149]
	Hemp fiber/eggshell	Epoxy	The hybrid polymer composites were prepared by using hemp fiber and eggshell particles using varying proportions of fillers at 0.25%, 0.5 %, and 1.0%. The mechanical results demonstrated that adding fiber to epoxy resin improved its load-bearing properties. Adding up to 0.5% eggshell as a filler enhanced the composite’s thermal stability.	Inbakumar and Ramesh (2018) [150]

## Data Availability

No new data were created or analyzed in this study. Data sharing is not applicable to this article.
